# Effects of a ciliary neurotrophic factor (CNTF) small-molecule peptide mimetic in an in vitro and in vivo model of CDKL5 deficiency disorder

**DOI:** 10.1186/s11689-024-09583-4

**Published:** 2024-11-26

**Authors:** Nicola Mottolese, Manuela Loi, Stefania Trazzi, Marianna Tassinari, Beatrice Uguagliati, Giulia Candini, Khalid Iqbal, Giorgio Medici, Elisabetta Ciani

**Affiliations:** 1 Department of Biomedical and Neuromotor Science, University of Bologna, Piazza Di Porta San Donato 2, 40126 Bologna, Italy; 2 Department of Neurochemistry, Inge Grundke-Iqbal Research Floor, New York State Institute for Basic Research in Developmental Disabilities, Staten Island, NY 10314 USA; 3 Phanes Biotech Inc, Malvern, PA 19355 USA

**Keywords:** *Cdkl5* KO mice, CNTF, Brain development, Dendritic pathology, Neuronal survival, Neuroinflammation

## Abstract

**Background:**

Mutations in the X-linked *CDKL5* gene underlie a severe epileptic encephalopathy, CDKL5 deficiency disorder (CDD), characterized by gross motor impairment, autistic features and intellectual disability. Absence of Cdkl5 negatively impacts neuronal proliferation, survival, and maturation in in vitro and in vivo models, resulting in behavioral deficits in the *Cdkl5* KO mouse. While there is no targeted therapy for CDD, several studies showed that treatments enabling an increase in brain BDNF levels give rise to structural and behavioral improvements in *Cdkl5* KO mice. P021, a tetra-peptide derived from the biologically active region of the human ciliary neurotrophic factor (CNTF), was found to enhance neurogenesis and synaptic plasticity by promoting an increase in BDNF expression in preclinical models of brain disorders, such as Alzheimer’s disease and Down syndrome, resulting in a beneficial therapeutic effect. Considering the positive actions of P021 on brain development and cognition associated with increased BDNF expression, the present study aimed to evaluate the possible beneficial effect of treatment with P021 in an in vitro and in vivo model of CDD.

**Methods:**

We used SH-*CDKL5*-KO cells as an in vitro model of CDD to test the efficacy of P021 on neuronal proliferation, survival, and maturation. In addition, both young and adult *Cdkl5* KO mice were used to evaluate the in vivo effects of P021, on neuroanatomical and behavioral defects.

**Results:**

We found that P021 treatment was effective in restoring neuronal proliferation, survival, and maturation deficits, as well as alterations in the GSK3β signaling pathway, features that characterize a human neuronal model of CDKL5 deficiency. Unexpectedly, chronic in vivo P021 treatment failed to increase BDNF levels and did not improve neuroanatomical defects in *Cdkl*5 KO mice, resulting in limited behavioral benefit.

**Conclusions:**

At present, it remains to be understood whether initiating the treatment prenatally, or prolonging the duration of treatment will be necessary in order to achieve similar results in vivo in CDD mice to those obtained in vitro.

**Supplementary Information:**

The online version contains supplementary material available at 10.1186/s11689-024-09583-4.

## Background

Cyclin-dependent kinase-like 5 (CDKL5) deficiency disorder (CDD) is an X-linked brain disorder caused by pathogenic variants in the *CDKL5* gene [1–3]. *CDKL5* encodes a serine-threonine kinase that is highly expressed in the developing and adult brain [2]. CDD patients suffer from severe neurodevelopment disorders, including early infantile epileptic encephalopathy, autism spectrum disorders, and intellectual disability [4, 5]. Despite being rare, affecting up to 1:40,000–60,000 live births [4, 6], CDD is among the most common genetic causes of severe epilepsy in childhood [7]. Presently, there is no targeted therapy for CDD that is able to address the underlying problems of the disorder.

To study the underlying mechanism of the pathogenesis of CDD, multiple mouse models were generated [8–12]. These models are characterized by phenotypes that mimic several clinical CDD manifestations [8–10, 13, 14], including severe learning and memory impairments, autistic-like behaviors, and motor stereotypies. The behavioral deficits are associated with neuroanatomical alterations, including defects in neuronal architecture, synaptogenesis and connectivity [9, 13–18], decreased survival of newborn cells in the dentate gyrus and of CA1 pyramidal neurons [17, 19, 20], and an increased status of microglia activation [21, 22]. In vivo CDD models have been extensively used to investigate possible therapeutic approaches for CDD [13, 15, 19, 22–30]. Among these, neurotrophic factors, such as BDNF and IGF-1, by virtue of their neurogenic and neurotrophic activities, were found to have positive effects on the brain phenotypes of *Cdkl5* KO mice [15, 22, 29, 30]*.* In particular, boosting the BDNF/TrkB pathway with a TrkB agonist [30] or increasing BDNF expression by voluntary exercise [31] improves memory performance of *Cdkl5* KO mice.

The neuropoietic cytokines, such as ciliary neurotrophic factor (CNTF) and leukemia inhibitory factor, are also considered members of the neurotrophic factor family [32–34]. CNTF is the most extensively studied neuropoietic cytokine, and its neuroprotective effects as well as its pivotal role in adult hippocampal neurogenesis, and in neural stem cell differentiation are well established [35–38]. However, like other neurotrophic factors, the clinical therapeutic usage of CNTF is restricted due to its limited blood–brain barrier (BBB) permeability, poor plasma stability and unsuitable pharmacokinetics, and unwanted systemic effects [39]. To bypass these limitations in developing CNTF based drugs for brain disorders, a CNTF small-molecule peptide mimetic, Peptide 021 (P021: Ac-DGGLAG-NH2), was generated from a 4-mer CNTF peptide by the addition of adamantylated glycine at the C-terminus [40, 41]. Interestingly, prenatal to early postnatal treatment with P021 rescued developmental delay in pups and hippocampus dependent memory impairments in adult life in a mouse model of Down syndrome (DS), the Ts65Dn mouse [42], suggesting that providing CNTF neurotrophic support in a critical period of brain development can be an effective therapeutic strategy for developmental disability in DS. P021 was shown to ameliorate learning and memory deficits in animal models of aging and Alzheimer’s disease (AD) [43, 44], and to rescue cognitive impairment in rodent models of DS and AD by enhancing transcription and expression of BDNF [42, 43].

Considering the positive actions of P021 on brain development and cognition [42–44], associated with increased BDNF expression [42, 43], we deemed it intriguing to evaluate whether treatment with P021 might have a positive impact in the context of CDD. Here, we examined the potential of treatment with P021 to rescue CDD-related phenotypes in in vitro and in vivo models of CDD.

## Materials and Methods

### Design and synthesis of P021

The peptidergic compound P021 (Ac-DGGLAG-NH2; molecular weight: 578.3) used in this work derived from the laboratory of Prof. Khalid Iqbal (New York State Institute for Basic Research in Developmental Disabilities, USA). This compound corresponds with the biologically active region of human ciliary neurotrophic factor (CNTF; amino acid residues 148–151) to which adamantylated glycine was added to increase its stability and lipophilicity [40, 45]. The peptide was synthesized and purified by reverse phase HPLC to ~ 96% purity, as previously described [41].

### Cell Lines, Treatments and Measurements

Human neuroblastoma cell line SH-SY5Y, obtained from The European Collection of Authenticated Cell Cultures (Sigma-Aldrich, Saint Louis, MO, USA) and the *CDKL5* knockout (KO) SH-SY5Y neuroblastoma cell line (SH-*CDKL5*-KO; [20]) were maintained in Dulbecco modified Eagle medium (DMEM, Thermo Fisher Scientific, Waltham, MA, USA) supplemented with 10% heat-inactivated FBS, 2 mM of L-glutamine, and antibiotics (penicillin, 100 U/ml; streptomycin, 100 μg/ml; Thermo Fisher Scientific, Waltham, MA, USA), in a humidified atmosphere of 5% of CO_2_ at 37 °C. Cell medium was replaced every 3 days and the cells were sub-cultured once they reached 90% confluence.

#### *P021 *in vitro* Treatment*

Cells were plated onto poly-D-lysine-coated slides in a 6-well plate at a density of 2.5 × 10^5^ cells per well in culture medium. The day after, cells were exposed to P021 (1 μM or 10 μM; stock solution 1 mM in deionized water) or vehicle (deionized water) for 24 h.

#### Retinoic Acid Induced Differentiation

For differentiation analyses, cells were plated onto poly-D-lysine-coated slides in a 6-well plate at a density of 1 × 10^5^ cells per well in culture medium. Two hours after cell plating, retinoic acid (RA; Sigma-Aldrich, Saint Louis, MO, USA) was added to the medium at 10 μM final concentration each day for 5 days. Cells were co-treated with P021 (10 μM) or vehicle (deionized water) every day.

#### Image Acquisition

Phase-contrast or fluorescence images were taken with an Eclipse TE 2000-S microscope equipped with a DS-Qi2 digital SLR camera (Nikon Instruments, Tokyo, Japan). Images were taken from random microscopic fields (10 for each coverslip).

#### Mitotic and Apoptotic index

To assess the number of mitotic and apoptotic cells, cultures were fixed in a 4% paraformaldehyde solution at 37 °C for 30 min and nuclei were stained with Hoechst 33342 (Sigma-Aldrich, Saint Louis, MO, USA). The number of mitotic cells was assessed by manually counting the cells in prophase (chromosomes are condensed and visible), metaphase (chromosomes are lined up at the metaphase plate), and anaphase/telophase (chromosomes are pulled toward and arrive at opposite poles) and expressed as a percentage of the total number of cells. Apoptotic cell death was assessed by manually counting the number of pyknotic nuclei and apoptotic bodies and was expressed as a percentage of the total number of cells.

#### Analysis of Neurite Outgrowth

Neurite outgrowth was measured using the image analysis system Image Pro Plus software version 4.5 (Media Cybernetics, Silver Spring, MD, USA). Only cells with neurites that were longer than one cell body diameter were considered as neurite-bearing cells. In each experiment, a total of 900 cells was analyzed. All experiments were performed at least three times. The total length of neurites was divided by the total number of cells counted in the areas.

### Colony

The mice used in this work were the *Cdkl5* KO strain in the C57BL/6N background developed in [9] and backcrossed in C57BL/6 J for three generations. For the present study, mice were produced by crossing *Cdkl5* KO heterozygous females (+ / −) with wild-type (+ /Y) males and they were genotyped using PCR of genomic DNA as previously described [9]. Littermate controls were used for all experiments. The day of birth was designated as postnatal day zero (P0), and animals with 24 h of age were considered as 1-day-old animals (P1). After weaning (P21-23), mice were housed 3 to 5 per cage and maintained in a temperature- (23 °C) and humidity-controlled environment with a standard 12 h light/dark cycle, and provided with mouse chow and water ad libitum. The animals’ health and comfort were controlled by the veterinary service. All the experiments were conducted in accordance with the Italian and European Community law for the use of experimental animals and with the approval of the National Bioethical Committee (approval number: n° 184/2022-PR). All efforts were made to minimize animal suffering and to reduce the number of animals used. Experiments were carried out on a total of 39 *Cdkl5* KO male mice (*Cdkl5* + /Y, *n* = 17; *Cdkl5* − /Y, *n* = 22).

### In Vivo Experimental Protocol

#### Chronic oral P021 treatment

Starting from postnatal day 21 (P21), *Cdkl5* − /Y mice were treated orally with a P021 diet for 70 days. Treatment was administered as 60 nmol peptide/g formulated diet (Research Diets, New Brunswick, NJ, USA). On average, each mouse consumed ~ 2.7 g diet/day. As a control, a group of wild-type (+ /Y) and *Cdkl5* − /Y mice received the same diet but without the peptide. On the forty-ninth day of treatment (P70), animals from all the experimental groups received a single intraperitoneal (i.p.) injection (150 µg/g body weight) of BrdU (5-bromo-2-deoxyuridine; Sigma-Aldrich, Saint Louis, MO, USA). Body weight of the mice was monitored every eight days. Animals were behaviorally tested from P74 to P89. The day after completion of the behavioral task (P90), animals were sacrificed and brain tissues were collected for histological and Western blot analyses.

#### Chronic P021 administration through intraperitoneal injections

Four-month-old *Cdkl5* − /Y male mice were intraperitoneally injected with P021 (750 nmol/mouse in saline) daily for 30 days. As a control, a group of wild-type (+ /Y) and *Cdkl5* − /Y mice were injected with vehicle (saline). Each animal received four successive i.p. injections of BrdU (5-bromo-2'-deoxyuridine; 150 µg/g body weight) at 2 h intervals (at approximately 10:00, 12:00, 14:00, and 16:00 h) on the first experimental day. Twenty-four hours after the last P021 administration, mice were sacrificed and brain tissues were collected for histological and Western blot analyses. Body weight of the mice was monitored every eight days.

### Behavioral Assays

After 53 days of P021 oral treatment, the animals were behaviorally tested with a sequence of tests, arranged to minimize the effect of one test influencing the subsequent evaluation of the next, and mice were allowed to recover for 1 day between different tests. All behavioral studies and analyses were performed blinded to genotype and treatment. Mice were allowed to habituate to the testing room for at least 1 h before the test, and testing was performed at the same time of day.

#### Marble Burying

The marble burying test was performed by placing animals individually in a home-cage-like environment with 5 cm of unscented standard bedding material and 20 marbles (14.3 mm in diameter) arranged in a 4 × 5 matrix, and were left undisturbed for 30 min. The number of marbles that were at least two-thirds buried at the end of the trial was counted.

#### Nesting

Nest building ability was evaluated as proposed by Deacon [46]. Animals were placed in individual cages with standard bedding, and a standard piece of paper towel (23 cm × 23 cm) was provided. The nests were independently assessed at 24 h by two operators using the following scoring system: 0—no nest, 1—primitive flat nest (pad-shaped, consisting of a flat paper tissue which slightly elevates a mouse above the bedding), 2—more complex nest (including warping and biting the paper towel), 3—complex accurate cup-shaped nests (with shredded paper interwoven to form the walls of the cup), and 4—complex hooded nest, with walls forming a ceiling so the nest becomes a hollow sphere with one opening.

#### Hind-Limb Clasping

Animals were suspended by their tail for 2 min and hind-limb clasping time was assessed from video recordings. A clasping event is defined by the retraction of hind-limbs into the body and toward the midline.

#### Accelerating Rotarod Assay

Before the first test session, animals were briefly trained at a constant speed of 5 rpm on the rotarod apparatus (Ugo Basile, Gemonio, Italy) for 30 s. Thirty minutes later, testing was performed at an accelerating linear speed (5–35 rpm within 270 s + 30 s max speed). Four testing trials, with an intertrial interval of 1 h, were performed. The latency to fall from the rotating rod and the number of passive rotations (rotation in which the mouse does not perform any coordinated movement but is passively transported by the rotating apparatus) were recorded for each trial.

#### Catalepsy Bar Test

The bar was set at a height of 6 cm. Mice were gently positioned, by placing both fore-limbs on the bar and their hind-limbs on the floor. The time needed for the mice to remove both paws from the bar was measured using a stopwatch.

#### Open Field

In order to assess locomotion, animals were placed in the center of a square arena (50 × 50 cm) and their behavior was monitored for 20 min using a video camera placed above the center of the arena. Distinct features of locomotor activity, including total distance traveled, average locomotion velocity, and the time spent in the central and peripheral zones, were scored by EthoVision 15XT software (Noldus Information Technology, Wageningen, The Netherlands). The test chambers were cleaned with 70% ethanol between test subjects.

#### Morris Water Maze

Hippocampal-dependent spatial learning and memory was assessed using the Morris water maze (MWM). Mice were trained to locate a hidden escape platform in a circular pool. The apparatus consisted of a circular water tank (1 m in diameter, 50 cm high) with a transparent round escape platform (10 cm^2^) placed in a fixed position. The tank was filled with tap water at a temperature of 22 °C up to 0.5 cm above the top of the platform, and the water was made opaque with milk. In the experimental room, intra-maze and extra-maze visual cues were placed to enable spatial orientation. Mouse behavior was automatically videotracked (EthoVision 3.1; Noldus Information Technology, Wageningen, The Netherlands). During training, each mouse was subjected to either 1 swimming session of 4 trials (day 1) or 2 sessions of 4 trials per day (days 2–5), with an intersession interval of 1 h (acquisition phase). Mice were allowed to search for the platform for up to 60 s. If a mouse did not find the platform, it was gently guided to it and allowed to remain there for 15 s. During the intertrial interval (15 s), mice were placed in an empty cage. The latency to find the hidden platform was used as a measure of learning. Twenty-four hours after the last acquisition trial, on day 6, the platform was removed and a probe test was run. Animals were allowed to search for the platform for up to 60 s. The latency of the first entrance into the former platform area was employed as measures of retention of acquired spatial preference. During the learning phase, the average and maximum swim speeds were also analyzed.

#### Passive Avoidance

For the passive avoidance task, a memory task that involves contributions from both the hippocampus and amygdala, the equipment consisted of a tilting-floor box (47 × 18 × 26 cm) divided into 2 compartments (lit and dark) by a sliding door, and a control unit that incorporated a shocker (Ugo Basile, Gemonio, Italy). Upon entering the dark compartment, mice received a brief mild foot shock (0.4 mA for 3 s) and were removed from the chamber after a 15-s delay. After a 24-h retention period, mice were returned to the illuminated compartment, and the latency to re-enter the dark chamber was measured, up to 360 s. The chambers were cleaned with 70% ethanol between testing of one subject and another.

### Histological and Immunohistochemistry Procedures

Animals were anesthetized with isoflurane (2% in pure oxygen) and sacrificed through cervical dislocation. Brains were quickly removed and cut along the midline. Left hemispheres were Golgi-stained or quickly frozen and used for Western blot analyses. Right hemispheres were fixed by immersion in 4% paraformaldehyde in 0.1 M phosphate-buffered saline (PBS) for 48 h, kept in 15–20% sucrose for an additional 24 h, frozen with dry ice, and stored at -80 °C. Right hemispheres were then cut with a freezing microtome (Microm GmbH, Walldorf, Germany) into 30-μm-thick coronal sections, which were serially collected in 96-well plates containing a solution composed of 30% glycerol, 30% ethylene glycol, 0.02% sodium azide in 0.1 M PBS, and then processed for immunohistochemistry procedures.

#### Immunofluorescence staining

One out of every eight free-floating sections from the hippocampal formation was incubated overnight at 4 °C with one of the following primary antibodies: rabbit polyclonal anti-AIF-1 antibody (1:300; Thermo Fisher Scientific, Waltham, MA, USA), mouse monoclonal anti-NeuN antibody (1:250; Merck Millipore, Burlington, MA, USA), or rabbit polyclonal anti-DCX antibody (1:300; Thermo Fisher Scientific, Waltham, MA, USA). The following day, the sections were incubated for 2 h at room temperature with a Cy3-conjugated anti-rabbit IgG secondary antibody (1:200; Jackson ImmunoResearch Laboratories, West Grove, PA, USA) for AIF-1 and DCX immunohistochemistry, and with a Cy3-conjugated anti-mouse IgG secondary antibody (1:200; Jackson ImmunoResearch Laboratories, West Grove, PA, USA) for NeuN immunohistochemistry. Nuclei were counterstained with Hoechst 33342 (Sigma-Aldrich, Saint Louis, MO, USA).

For BrdU immunofluorescence, one out of every eight free-floating sections from the hippocampal formation was denatured in 2 N HCl for 30 min at 37 °C, and then incubated overnight at 4 °C with a rat monoclonal anti-BrdU antibody (1:200; Abcam, Cambridge, UK). The following day, the sections were incubated for 2 h at room temperature with a Cy3-conjugated anti-rat IgG secondary antibody (1:200; Jackson ImmunoResearch Laboratories, West Grove, PA, USA). Nuclei were counterstained with Hoechst 33342 (Sigma-Aldrich, Saint Louis, MO, USA).

Fluorescent images were acquired using an Eclipse TE 2000-S microscope equipped with a DS-Qi2 digital SLR camera (Nikon Instruments, Tokyo, Japan).

#### Golgi staining

Left hemispheres were Golgi-stained using the FD Rapid Golgi StainTM Kit (FD Neuro Technologies, Columbia, MD, USA). Briefly, hemispheres were immersed in the impregnation solution containing mercuric chloride, potassium dichromate, and potassium chromate, and stored at room temperature in the dark for 3 weeks. Hemispheres were then cut with a cryostat (Histo-Line Laboratories, Pantigliate, Italy) into 100-µm-thick coronal sections, which were directly mounted onto Superfrost® Plus Microscope Slides (Thermo Fisher Scientific, Waltham, MA, USA) and air-dried at room temperature for 1 day. After drying, sections were rinsed with distilled water, stained in the developing solution of FD Rapid Golgi StainTM Kit (FD NeuroTechnologies, Columbia, MD, USA), and coverslipped with DPX mounting medium (Sigma-Aldrich, Saint Louis, MO, USA). A light microscope (Leica Mycrosystems, Wetzlar, Germany) equipped with motorized stage, focus control system, and color digital camera (Coolsnap-Pro; Media Cybernetics, Rockville, MD, USA) were used to acquire bright field images.

### Measurements

#### Cell density

The number of BrdU-, and DCX-positive cells were counted in the subgranular and granular zone of the dentate gyrus of the hippocampus and expressed as number of cells/100 µm. The density of Hoechst-positive nuclei and neurons (NeuN-positive cells) in the CA1 field of the hippocampus were manually counted and expressed as cells/mm^3^.

#### Morphometric microglial cell analysis

Starting from 20 × magnification images of AIF-1-stained cortical slices, microglial cell body size was manually drawn using the measurement function of the Image-Pro Plus software (Media Cybernetics, Rockville, MD, USA) and expressed in μm^2^. Approximately 120 microglial cells were analyzed from each sample.

#### Dendritic Spine Number and Morphology

In Golgi-stained sections, dendritic spines of hippocampal pyramidal neurons were visualized with a 100 × oil immersion objective lens. Dendritic spine density was measured by manually counting the number of dendritic spines on the basal dendrites of hippocampal pyramidal neurons and was expressed as total number of spines per 10 μm. Based on their morphology, dendritic spines can be divided into two different categories that reflect their state of maturation: immature spines (filopodium-like, thin- and stubby-shaped) and mature spines (mushroom- and cup-shaped). The number of mature spines was counted and expressed as a percentage. About 100–150 spines from 15 to 20 dendrites, derived from 10 to 20 neurons, were analyzed per condition.

### Western Blotting

To score the amount of phospho-proteins in cell culture we used a rapid protein extraction method [47] that significantly reduces the risk of protein degradation and modifications that may occur during harvesting and cell lysis. SH-SY5Y and SH-*CDKL5*-KO cells were plated in a 6-well plate at a density of 2.5 × 10^5^ cells per well in culture medium. The following day, cells were exposed to 10 μM P021 or vehicle (deionized water). Six hours after the treatment the culture medium was removed from the plate and adherent cells were washed with ice cold PBS and directly lysed in the plate by adding 100 μl of denaturing Laemmli loading buffer 2x. Samples were collected, boiled in a mixing heating block for 10 min at 99 °C, and equal amounts (20 μl) of each sample were subjected to electrophoresis on a Bolt^TM^ 4–12% Bis–Tris Plus gel (Life Technologies Corporation, Carlsbad, CA, USA).

Tissue samples from the hippocampus of vehicle-treated *Cdkl5* + /Y and *Cdkl5* − /Y mice and of P021-treated *Cdkl5* − /Y mice, were lysed in ice-cold RIPA buffer (50 mM Tris–HCl, pH 7.4, 150 mM NaCl, 1% Triton-X100, 0.5% sodium deoxycholate, 0.1% SDS) supplemented with 1 mM PMSF, and with 1% protease and phosphatase inhibitor cocktail (Sigma-Aldrich, Saint Louis, MO, USA). Protein concentration for both cell and tissue extracts was determined using the Bradford method [48]. Equivalent amounts of protein (50 µg) were subjected to electrophoresis on a Bolt^TM^ 4–12% Bis–Tris Plus gel and transferred to a Hybond ECL nitrocellulose membrane (GE Healthcare Bio-Science, Piscataway, NJ, USA).

The following primary antibodies were used: rabbit polyclonal anti-BDNF (1:500; Santa Cruz Biotechnology, Dallas, TX, USA), rabbit polyclonal anti-phospho-GSK3β (Ser9; 1:1000; Cell Signaling Technology, Danvers, MA, USA), rabbit polyclonal anti-GSK3β (1:1000; Cell Signaling Technology, Danvers, MA, USA), rabbit polyclonal anti-phospho-Akt (Ser473; 1:1000; Cell Signaling Technology, Danvers, MA, USA), rabbit polyclonal anti-Akt (1:1000; Cell Signaling Technology, Danvers, MA, USA), mouse monoclonal anti-Vinculin (7F9; 1:500; Santa Cruz Biotechnology, Dallas, TX, USA), and rabbit polyclonal anti-GAPDH (1:5000; Sigma-Aldrich, Saint Louis, MO, USA). An HRP-conjugated goat anti-rabbit IgG secondary antibody (1:5000; Jackson ImmunoResearch Laboratories, West Grove, PA, USA) and an HRP-conjugated goat anti-mouse IgG secondary antibody (1:5000; Jackson ImmunoResearch Laboratories, West Grove, PA, USA) were used. Densitometric analysis of digitized Western blot images was performed using Chemidoc™ XRS + Imaging System and the Image Lab™ Software (Bio-Rad, Hercules, CA, USA). This software automatically highlights any saturated pixels of Western blot images in red. Images acquired with exposure times that generated protein signals out of a linear range were not considered for quantification. Western blot analyses were performed on protein extracts of multiple samples per experimental group: three to five biological replicates for the cell lines or three to six for animals. Repeated measurements of the same samples were performed by running from two to four independent gels. The signal of one sample (internal control) was used to perform a relative analysis of the antigen expression of each sample on the same gel. We considered the control signal as 100 and assigned a value to the other sample as a percentage of the control. Data analysis was performed by averaging the signals obtained in two to four gels for each individual sample.

### Statistical Analysis

Statistical analysis was performed using GraphPad Prism 8.0.1 (GraphPad Software, Boston, MA, USA). Values are expressed as means ± standard error (SEM). The significance of results was obtained using Student’s t-test, an ordinary one-way or two-way analysis of variance (ANOVA) or a two-way repeated measurement (RM) ANOVA followed by Fisher’s LSD post hoc test, as specified in the figure legends. A probability level of p < 0.05 was considered statistically significant. The confidence level was taken as 95%.

## Results

### Treatment with P021 restores proliferation, survival and neuronal maturation of a human cellular model of CDKL5 deficiency

**Fig. 1:**
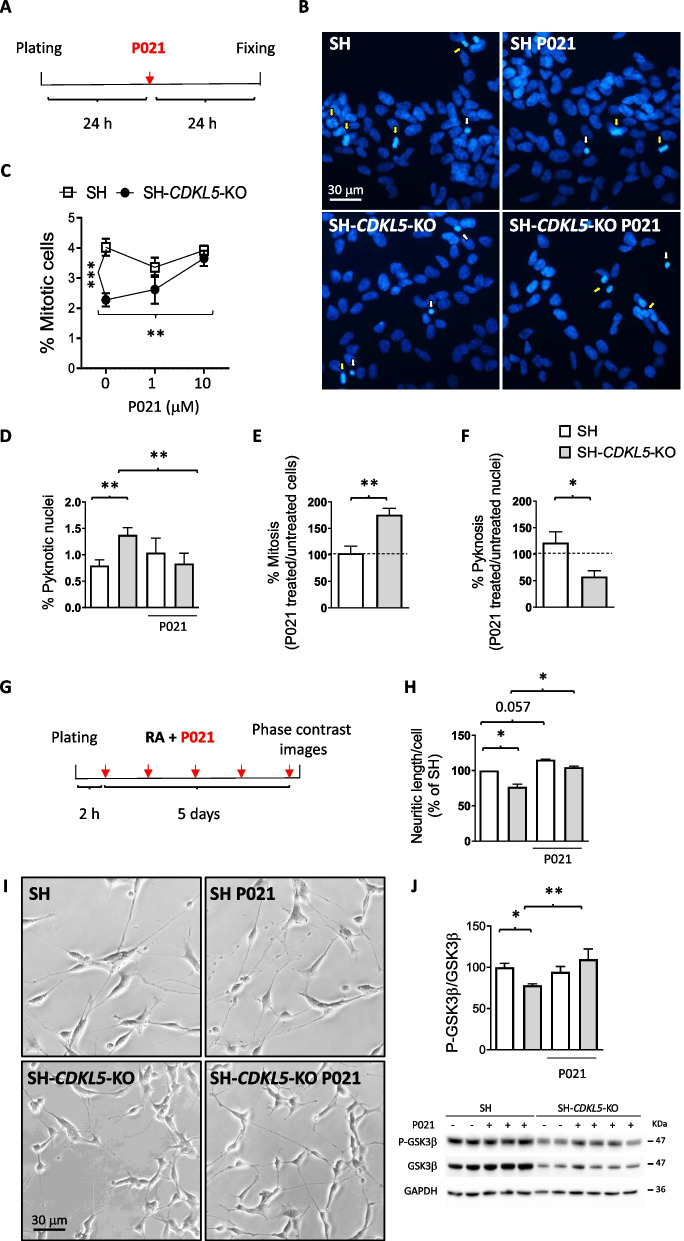
Effect of P021 treatment on cell proliferation, survival, and differentiation in SH-*CDKL5*-KO cells. **A** Schematic representation of the experimental design to assess proliferation and survival in SH-SY5Y and SH-*CDKL5*-KO cells. **B** Representative fluorescence images of Hoechst-stained nuclei (white arrows indicate pyknotic nuclei, yellow arrows indicate mitotic nuclei) of untreated and P021 (10 µM)-treated SH-SY5Y (SH) and SH-*CDKL5*-KO cells. **C** Percentage of mitotic cells in untreated (0 µM) and P021 (1 µM and 10 µM)-treated SH-SY5Y and SH-*CDKL5*-KO cells 24 h after the treatment administration.** D** Percentage of pyknotic nuclei in untreated and P021 (10 µM)-treated SH-SY5Y and SH-*CDKL5*-KO cells. **E** Percentage of mitotic cells in SH-SY5Y and SH-*CDKL5*-KO cells, expressed as ratio of P021-treated to untreated cells. **F** Percentage of pyknotic nuclei in SH-SY5Y and SH-*CDKL5*-KO cells, expressed as ratio of P021-treated to untreated cells. **G** Schematic representation of the experimental design to assess differentiation in SH-SY5Y and SH-*CDKL5*-KO cells. Cells were treated daily with retinoic acid (RA; 10 μM) and P021 (10 μM) for 5 days. **H** Quantification of neurite outgrowth after RA-induced differentiation with or without P021 treatment, in parental cell line (SH) and SH-*CDKL5*-KO cells, expressed as a percentage of the SH-SY5Y untreated condition. **I** Representative phase-contrast images of neurite outgrowth of RA-treated and RA/P021-treated SH-SY5Y (SH) and SH-*CDKL5*-KO cells. **J** Western blot analysis of phospho-GSK3β (Ser9; P-GSK3β) levels in protein extracts from untreated (SH *n* = 5; SH-*CDKL5*-KO *n* = 5) and P021-treated SH-SY5Y (SH *n* = 3) and SH-*CDKL5*-KO (*n* = 4) cells. The histogram shows P-GSK3β protein levels normalized to total GSK3β levels. Data are expressed as a percentage of vehicle-treated SH-SY5Y cells. Examples of immunoblot for P-GSK3β, GSK3β, and GAPDH in the lower panel. Values are represented as means ± SEM of 3 independent experiments. * p < 0.05, ** p < 0.01, *** p < 0.001. Fisher’s LSD test after two-way ANOVA for data set in (**C**, **D**, **H, J**) and Student’s t-test for data set in (**E**, **F**)

In order to investigate whether treatment with P021 rescued CDD-related phenotypes we used a human neuronal cell model of CDKL5 deficiency, the *CDKL5* knockout (KO) SH-SY5Y neuroblastoma cell line (SH-*CDKL5*-KO; [20]), which shows impaired cell proliferation and survival, and deficit in neuronal maturation [20]. The effect of P021 treatment on SH-*CDKL5*-KO cell proliferation (Fig. 1A) was evaluated as a percentage of mitotic nuclei visualized with Hoechst staining (Fig. 1B,C). As previously reported [20], SH-*CDKL5*-KO cells showed a reduced number of mitotic cells (Fig. 1B,C) compared to parental cells. We found a dose response increase in cell proliferation in P021-treated SH-*CDKL5*-KO cells, a proliferation that was completely recovered to the levels of the control SH-SY5Y condition at the P021 dose of 10 μM (Fig. 1C). Similarly, we found that treatment with P021 (10 μM) restored cell survival, evaluated as the fraction of pyknotic nuclei, in SH-*CDKL5*-KO cells (Fig. 1B,D). In control SH-SY5Y cells, treatment with P021 had no effect on cell proliferation and survival (Fig. 1E,F).

It is known that SH-SY5Y neuroblastoma cells can be differentiated by treatment with retinoic acid (RA) to obtain a more mature neuron-like phenotype, characterized by increased neuronal markers expression and neurite outgrowth [49, 50]. After treatment with RA for 5 days (Fig. 1G), differentiated SH-*CDKL5*-KO cells showed reduced neurite outgrowth in comparison with control SH-SY5Y cells (Fig. 1H,I). Treatment with P021 (10 µM) restored neuritic length in SH-*CDKL5*-KO cells to that of SH-SY5Y cells (Fig. 1H,I). Albeit to a lesser extent, treatment with P021 increased neuritic length in SH-SY5Y cells (Fig. 1H), confirming the positive effect of CNTF signaling in neuronal maturation.

### Treatment with P021 restores GSK3β signaling in a human cellular model of CDKL5 deficiency

There are different signaling pathways activated by the CNTF signaling that occurs through a tripartite complex of CNTF receptor α (CNTFRα), LIF β receptor (LIFR), and gp130 [38]. In neuroblastoma cells the CNTF-induced PI3K-Akt-GSK3β pathway is important for survival and neurite growth [51]. As previously reported [20], SH-*CDKL5*-KO cells showed lower phosphorylation levels of GSK3β protein compared to SH-SY5Y cells (Fig. 1J). Treatment with P021 restored P-GSK3β levels in SH-*CDKL5*-KO cells to control levels (Fig. 1J), whereas no significant differences in GSK3β phosphorylation were observed in P021-treated SH-SY5Y cells (Fig. 1J). No differences were found between SH-*CDKL5*-KO and parental cells in total levels of GSK3β (Fig. S1), and no effect of P021 treatment was detected (Fig S1).

### Effect of juvenile treatment with P021 on behaviors of *Cdkl5* KO mice

**Fig. 2:**
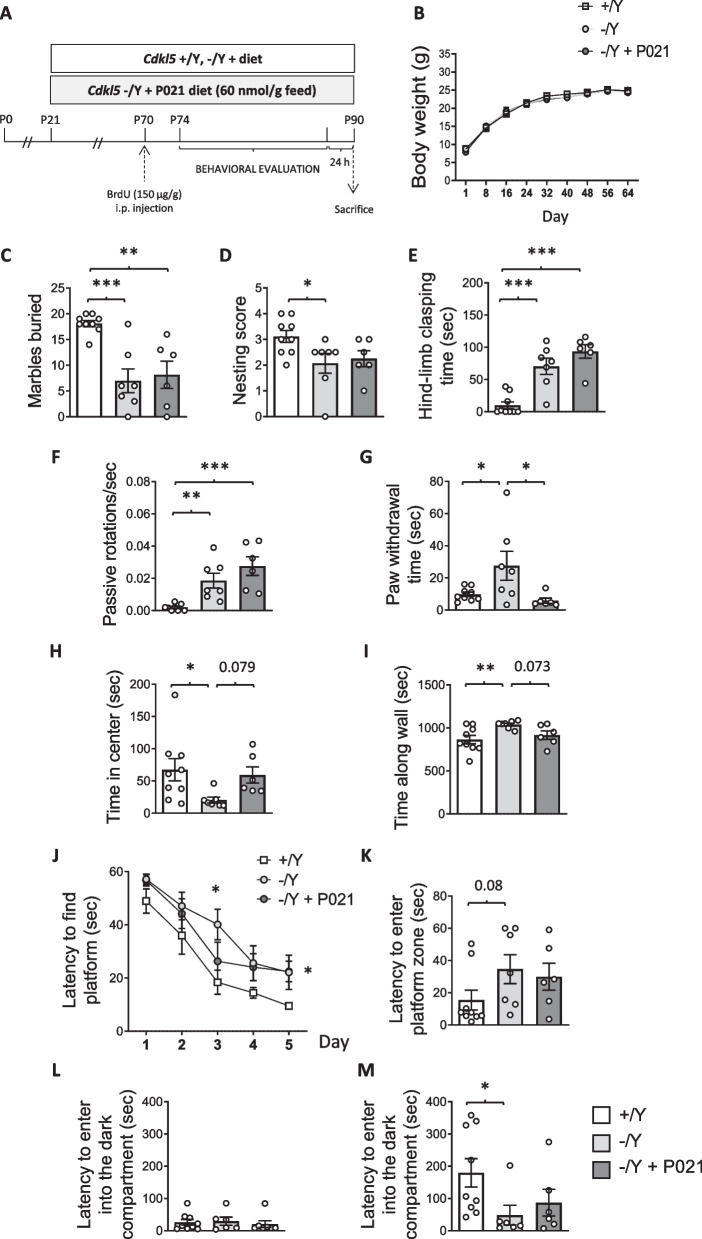
Effect of chronic oral P021 treatment on behavior in *Cdkl5* KO mice. **A** Experimental protocol. Starting from postnatal day 21 (P21), *Cdkl*5 − /Y mice were orally treated with the P021 diet, containing 60 nmol peptide/g formulated diet, for 70 days. As a control *Cdkl*5 − /Y and *Cdkl*5 + /Y mice were administered with the same diet but without P021. Animals from different experimental groups received a single intraperitoneal (i.p.) injection (150 μg/g body weight) of BrdU at P70 and were behaviorally tested starting from P74 to P89. Animals were sacrificed the day after completion of the behavioral task (P90). **B** Body weight in grams of vehicle-treated (+ /Y *n* = 9, − /Y *n* = 7) and P021-treated (− /Y + P021 *n* = 6) *Cdkl*5 male mice over the whole treatment period. **C**, **D** Autistic-like features in treated *Cdkl*5 − /Y mice. Number of marbles buried (**C**) and nest quality (**D**) of vehicle-treated *Cdkl*5 + /Y and − /Y mice and of P021-treated *Cdkl5* − /Y mice as in B. **E** Total amount of time spent hind-limb clasping during a 2-min interval in vehicle-treated *Cdkl*5 + /Y and − /Y mice and in P021-treated *Cdkl*5 − /Y mice as in B. **F** Frequency of passive rotations on the accelerating rotating rod in vehicle-treated *Cdkl*5 + /Y (*n* = 8) and − /Y (*n* = 7) mice and in P021-treated *Cdkl*5 − /Y mice (*n* = 6). **G** Amount of time taken to remove both paws from the bar in vehicle-treated *Cdkl*5 + /Y and − /Y mice and in P021-treated *Cdkl*5 − /Y mice as in B. **H**, **I** Total amount of time spent in the center (**H**), and along the walls (**I**) of the open field arena during the 20-min trial in vehicle-treated *Cdkl*5 + /Y and − /Y mice and in P021-treated *Cdkl*5 − /Y mice as in B.** J** Spatial learning (5-day learning period) assessed using the Morris water maze in vehicle-treated *Cdkl*5 + /Y and − /Y mice and in P021-treated *Cdkl*5 − /Y mice as in B. **K** Spatial memory on day 6 (probe test). Memory was assessed by evaluating the latency to enter the former platform zone. **L**, **M** Latency of entrance into the dark compartment of the passive avoidance apparatus during the training day (**L**) and the probe day (**M**) of the test in vehicle-treated *Cdkl*5 + /Y (*n* = 9) and − /Y (*n* = 7) mice and in P021-treated *Cdkl*5 − /Y mice (*n* = 6). Values are represented as means ± SEM. * p < 0.05, ** p < 0.01, *** p < 0.001. Fisher’s LSD test after one-way ANOVA for data set in (**C-I**, **K-M**) and after two-way RM ANOVA for data set in (**B**, **J**)

Based on the results obtained in vitro we sought to investigate whether in vivo treatment with P021 improves the neurodevelopmental alterations that characterize *Cdkl5* − /Y mice. We chronically treated *Cdkl5* − /Y mice with P021, given in the food diet at a concentration of 60 nmol/g feed [52, 53] (Fig. 2A)*,* starting from a juvenile age (postnatal day 21, P21), with the aim of starting the treatment with P021 in a time window that is important for brain development. Twenty days before sacrifice all mice were treated with BrdU to label proliferating cells. During the last two weeks of treatment, a battery of behavioral tests was carried out to evaluate treatment efficacy (Fig. 2A). A group of *Cdkl5* − /Y and wild-type (+ /Y) mice treated with the same diet but without P021 were used as controls for behavioral tests. No changes in terms of body weight were observed in P021-treated *Cdkl5* − /Y mice compared to age-matched wild-type (+ /Y) and *Cdkl5* − /Y mice (Fig. 2B), indicating that treatment with P021 did not affect animal growth or well-being.

Loss of Cdkl5 function in *Cdkl5* − /Y mice is associated with autistic-like (ASD-like) phenotypes, analyzed through home-cage social behaviors (marble burying and nest building ability) [27]. *Cdkl5* − /Y mice buried a significantly lower number of marbles and showed a reduced nest building ability compared to *Cdkl5* + /Y mice (Fig. 2C,D). No significant change was found in *Cdkl5* − /Y mice treated with P021. Similarly, no improvement in P021-treated *Cdkl5* − /Y mice was detected in terms of motor stereotypies, a common feature of CDD [54], evaluated as hind-limb clasping time (Fig. 2E). Motor coordination of *Cdkl5* KO mice was also assessed on an accelerating rotarod by evaluating the frequency of passive rotations (number of passive rotations/sec). Rotarod performance notably differed between *Cdkl5* + /Y and *Cdkl5* − /Y mice, with *Cdkl5* − /Y mice showing significantly more passive rotations (Fig. 2F). We detected no improvement in locomotion in *Cdkl5* − /Y mice after treatment with P021 (Fig. 2F).

Catalepsy bar tests are widely used to measure the failure, resulting from muscular rigidity or akinesia, to correct an imposed posture [55]. As previously reported [25] we found that *Cdkl5* − /Y mice spent significantly more time hanging onto the bar than did *Cdkl5* + /Y mice (Fig. 2G). Treatment with P021 restored this motor behavior in *Cdkl5* − /Y mice (Fig. 2G), suggesting that treatment improves muscular rigidity due to loss of Cdkl5.

To evaluate anxiety-like behavior, we performed an open-field test and compared the time spent in the center of the arena, and near the walls, among groups. We found that *Cdkl5* − /Y mice spent less time in the center and more time close to the wall of the arena compared to *Cdkl5* + /Y mice (Fig. 2H,I), suggesting increased anxiety. Treatment with P021 revealed a trend to decrease this anxiety (Fig. 2H,I). No difference in locomotor activity, assessed as distance traveled and mean velocity, was present among groups (Fig. S2A,B).

Hippocampus-dependent learning and memory were evaluated using the Morris Water Maze (MWM) and the passive avoidance test. A repeated ANOVA on escape latency during the MWM learning phase revealed a significant genotype effect [*F*_(2, 19)_ = 4.420, *p* < 0.0265]. In the learning phase, both *Cdkl5* − /Y and P021-treated *Cdkl5* − /Y mice showed a reduced ability to learn over time compared to *Cdkl5* + /Y mice (Fig. 2J). In the probe test, as previously reported [25, 56], *Cdkl5* − /Y mice showed an increased latency to enter the former platform zone (Fig. 2K). Treatment with P021 in *Cdkl5* − /Y mice showed no improvement (Fig. 2K). No difference in distance moved or swim speed was found among groups (Fig. S2C,D), indicating that the deficit of *Cdkl5* − /Y mice in the MWM was not caused by abnormalities in locomotor activity or coordination. In the passive avoidance test, while no difference in step-through latency was found on the first day (Fig. 2L), *Cdkl5* − /Y mice show a significantly decreased latency on the second day, indicating a defect in associative memory. P021-treated *Cdkl5* − /Y mice did not show a significant memory improvement (Fig. 2M).

### Effect of juvenile treatment with P021 on hippocampal neurogenesis, neuronal survival, and spine development in *Cdkl5* KO mice

**Fig. 3:**
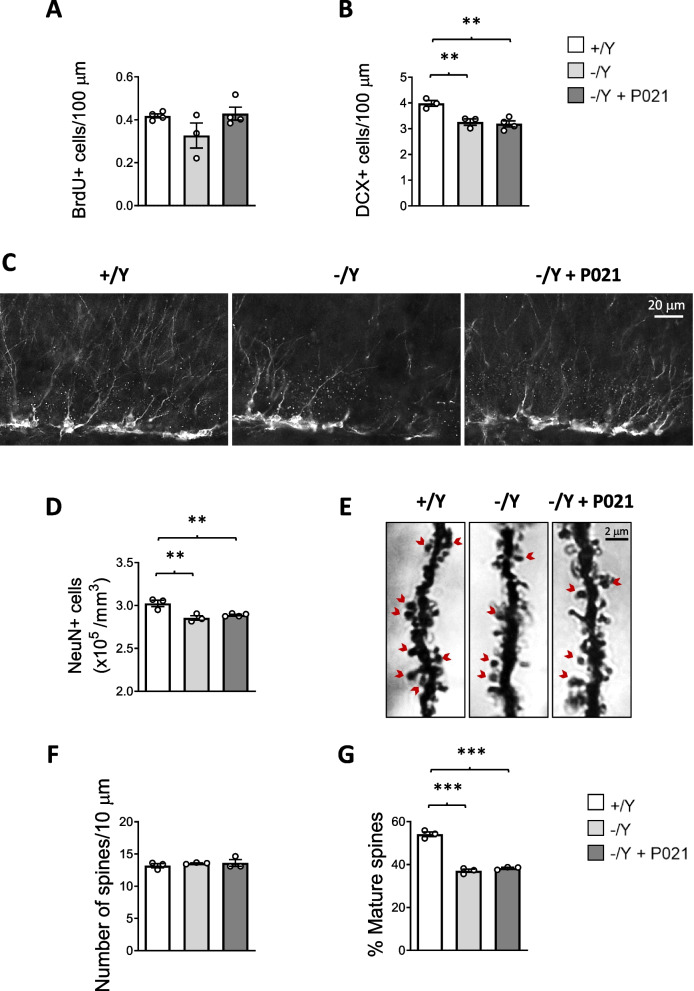
Effect of chronic oral P021 treatment on neuronal survival and dendritic spine maturation in the brain of *Cdkl5* KO mice. **A** Number of BrdU-positive cells in the subgranular zone (SGZ) of the dentate gyrus (DG) of hippocampal sections from vehicle-treated (+ /Y *n* = 4, − /Y *n* = 3) and P021-treated (− /Y + P021 *n* = 4) *Cdkl5* male mice. **B** Number of DCX-positive cells in the upper granular layer of the DG of hippocampal sections from vehicle-treated *Cdkl*5 + /Y (*n* = 3) and − /Y mice (*n* = 3) and from P021-treated *Cdkl*5 − /Y mice (*n* = 4). **C** Examples of hippocampal sections processed for DCX immunostaining of one animal from each experimental group. **D** Number of NeuN-positive cells in the CA1 field of hippocampal sections from vehicle-treated *Cdkl*5 + /Y and − /Y mice and from P021-treated *Cdkl*5 − /Y mice as in B. **E** Examples of Golgi-stained hippocampal pyramidal neurons of one animal from each experimental group; red arrows are examples of mature mushroom-shaped spines. **F** Dendritic spine density in hippocampal pyramidal neurons of vehicle-treated (+ /Y *n* = 3, − /Y *n* = 3) and P021-treated (-/Y + P021 *n* = 3) *Cdkl5* male mice. Data are expressed as number of spines per 10 µm. **G** Percentage of mature dendritic spines in relation to the total number of protrusions in hippocampal pyramidal neurons of *Cdkl*5 + /Y and *Cdkl*5 − /Y mice as in F. Values are represented as means ± SEM. ** p < 0.01, *** p < 0.001. Fisher’s LSD test after one-way ANOVA

Evidence has demonstrated that treatment with P021 boosts adult hippocampal neurogenesis and promotes neuronal survival in mouse models of AD [44, 53]. To establish the effect of treatment with P021 on hippocampal neurogenesis, we first evaluated the number of BrdU-positive cells in the hippocampal dentate gyrus (DG). As previously reported [31], no difference in the number of BrdU-positive cells was found between *Cdkl5* − /Y and *Cdkl5* + /Y mice (Fig. 3A); treatment with P021 had no effect on progenitor cell proliferation (Fig. 3A). In order to establish whether treatment with P021 affects progenitor cell survival in the DG, immunohistochemistry for doublecortin (DCX), a microtubule-associated protein expressed by differentiating (postmitotic) granule neurons, was performed. *Cdkl5* − /Y mice had a reduced number of new granule cells (DCX-positive cells) in comparison with *Cdkl5* + /Y mice (Fig. 3B,C), and treatment with P021 did not restore the survival of newborn cells (Fig. 3B,C).

To further assess the effect of P021 on neuron survival, we evaluated pyramidal neuron cell density in the CA1 layer of the hippocampus of treated *versus* untreated mice. *Cdkl5* − /Y mice showed a low number of pyramidal neurons in the CA1 layer compared to *Cdkl5* + /Y mice (Fig. 3D), and P021 did not improve the survival of pyramidal neurons in *Cdkl5* − /Y mice (Fig. 3D).

Similarly, spine maturation was not affected by P021 treatment. While no difference in spine density of Golgi-stained CA1 pyramidal neurons was observed in *Cdkl5* − /Y mice (Fig. 3E,F), *Cdkl5* − /Y mice had a reduced percentage of mature spines compared to wild-type mice (Fig. 3E,G). Treatment with P021 did not increase the number of mature spines in *Cdkl5* − /Y mice (Fig. 3E,G).

### Effect of juvenile treatment with P021 on microglia activation in *Cdkl5* KO mice

**Fig. 4:**
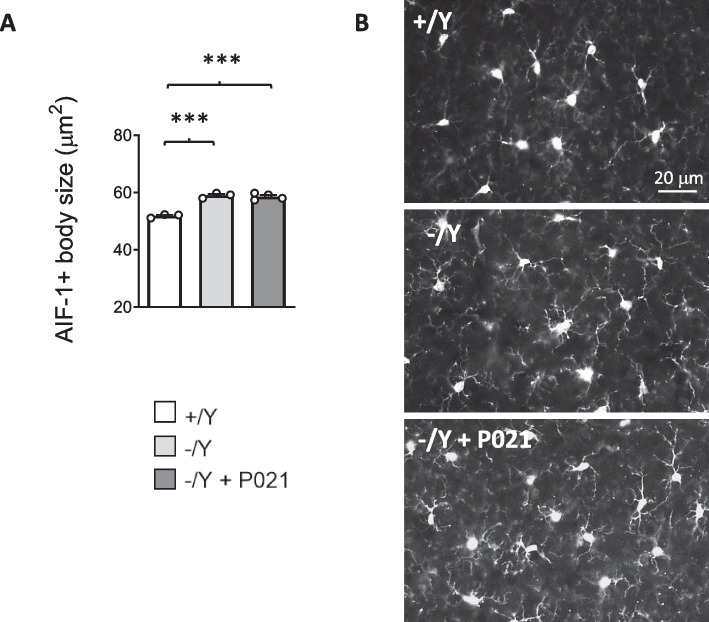
Effect of chronic oral P021 treatment on microglia activation in the brain of *Cdkl5* KO mice. **A** Mean cell body size of AIF-1-positive cells in cortical sections from vehicle-treated (+ /Y *n* = 3, − /Y *n* = 3) and P021-treated (− /Y + P021 n = 4) *Cdkl5* male mice. **B** Examples of cortical sections processed for AIF-1 immunostaining of a vehicle-treated wild-type (+ /Y) mouse, a vehicle-treated *Cdkl5* − /Y mouse, and a P021-treated *Cdkl5* − /Y mouse. Values are represented as means ± SEM. *** p < 0.001. Fisher’s LSD test after one-way ANOVA

*Cdkl5* KO mice are characterized by increased microglial activation [21, 22, 31]. Since it has been demonstrated that CNTF inhibits microglial activation [57], we explored whether treatment with P021 improves microglial cell status in the cortex of *Cdkl5* − /Y mice. No reversal of the inflammatory status, evaluated as a reduction in microglial soma size compared to the control levels, was present in P021-treated *Cdkl5* − /Y mice (Fig. 4A,B).

### Effect of juvenile treatment with P021 on BDNF expression in *Cdkl5* KO mice

Considering the finding that treatment with P021 increases BDNF levels in the brain of aged AD model mice (3xTg-mice, [42]), BDNF levels were analyzed in hippocampal extracts of P021-treated *Cdkl5* KO mice using Western blots. P021 treatment did not induce an increase in BDNF levels in *Cdkl5* − /Y mice (Fig. S3A,B). Accordingly, BDNF-mediated activation of TrkB-PI3K-Akt-GSK3β signaling was not increased in P021-treated *Cdkl5* − /Y mice (Fig. S3C,D).

### Effect of adult treatment with P021 on hippocampal development, microglia activation, and BDNF levels in *Cdkl5* KO mice

**Fig. 5:**
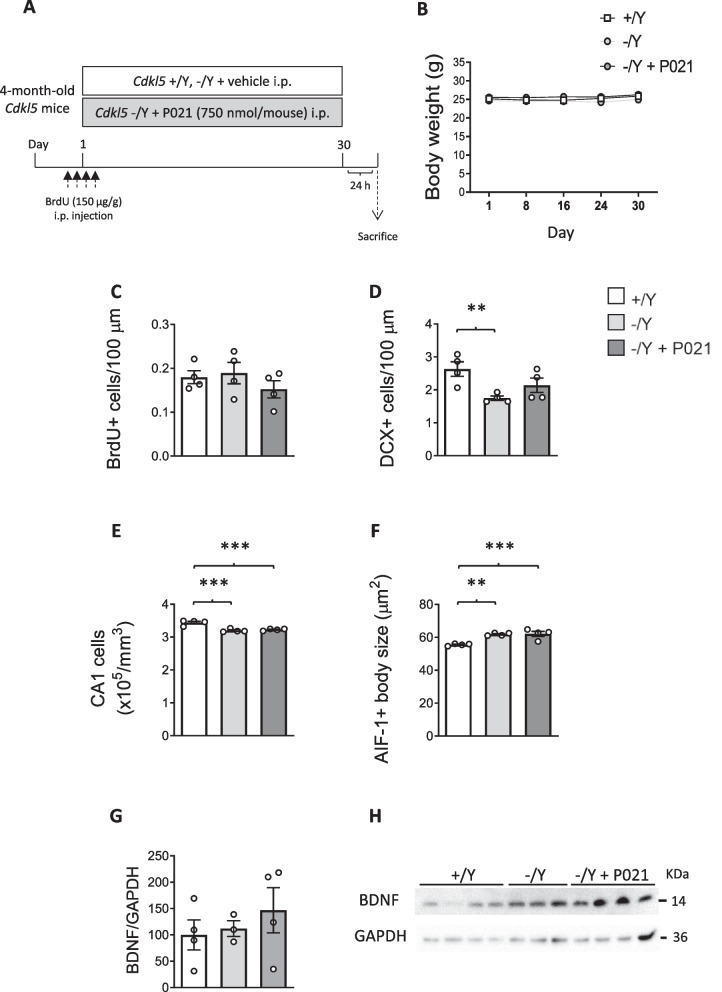
Effect of P021 administration through intraperitoneal injection in adult *Cdkl5* KO mice. **A** Experimental protocol. 4-month-old *Cdkl*5 + /Y mice were treated with vehicle, and *Cdkl*5 − /Y mice were treated with vehicle or P021 (750 nmol/mouse), administered via intraperitoneal injection (i.p.) every day for 30 days. Each animal received four successive i.p. injections of BrdU (5-bromo-2'-deoxyuridine; 150 µg/g body weight) at 2 h intervals on the first experimental day. Animals from different experimental groups were sacrificed 24 h after the last P021 injection. **B** Body weight in grams of vehicle-treated (+ /Y *n* = 8, − /Y *n* = 5) and P021-treated (− /Y + P021 n = 4) *Cdkl*5 male mice during the treatment period. **C** Number of BrdU-positive cells in the subgranular zone (SGZ) of the dentate gyrus (DG) of hippocampal sections from vehicle-treated (+ /Y *n* = 4, − /Y *n* = 4) and P021-treated (− /Y + P021 *n* = 4) *Cdkl5* male mice. **D** Number of DCX-positive cells in the upper layer of the DG of hippocampal sections from vehicle-treated *Cdkl*5 + /Y and − /Y mice and from P021-treated *Cdkl*5 − /Y mice as in C. **E** Number of Hoechst-positive cells in the CA1 field of hippocampal sections from vehicle-treated (+ /Y n = 4, − /Y n = 4) and P021-treated (− /Y + P021 n = 4) *Cdkl5* mice. **F** Mean cell body size of AIF-1-positive cells in cortical sections from vehicle-treated (+ /Y n = 4, − /Y n = 4) and P021-treated (− /Y + P021 n = 4) *Cdkl5* mice. **G, H** Western blot analysis of BDNF levels in hippocampal homogenates from vehicle-treated (+ /Y *n* = 4, − /Y *n* = 3) and P021-treated (− /Y + P021 *n* = 4) *Cdkl5* male mice. The histogram in G shows mature BDNF protein levels normalized to GAPDH protein levels. Examples of immunoblots from multiple biological replicates of each experimental condition in H. Data are expressed as a percentage of vehicle-treated wild-type (+ /Y) mice. Values are represented as means ± SEM. ** p < 0.01, *** p < 0.001. Fisher’s LSD test after two-way RM ANOVA for data set in (**B**) and after one-way ANOVA for data set in (**C-G**)

To exclude the possibility that the lack of effect of chronic treatment with P021 starting from juvenile age in *Cdkl5* KO mice depends on the P021 dose (60 nmol/g feed, corresponding to a 150 nmol/mouse/day) or on mouse age, we treated adult (4-month-old) *Cdkl5* − /Y mice with a higher dose of P021 (750 nmol/mouse), administered i.p. every day for 30 days. Mice were treated with 4 BrdU injections on the first day of treatment to label the entire pool of proliferating cells (Fig. 5A). Similar to juvenile animals, no changes in terms of body weight were observed in adult *Cdkl5* − /Y mice after treatment with P021 compared to age-matched vehicle-treated wild-type (+ /Y) and *Cdkl5* − /Y mice (Fig. 5B).

No effect of P021 treatment was found regarding hippocampal neurogenesis, evaluated as number of BrdU-positive cells (Fig. 5C), and number of DCX-positive cells (Fig. 5D) in *Cdkl5* − /Y mice. Similarly, no improvement in CA1 pyramidal neuron survival (Fig. 5E) or in cortical microglia overactivation (Fig. 5F) was present after P021 treatment. Again, similar to juvenile-treated *Cdkl5* KO mice, P021 treatment did not induce any significant effect on BDNF levels in adult *Cdkl5* − /Y mice (Fig. 5G,H).

## Discussion

Neurotrophic factors play a crucial role in neuronal differentiation, maturation, and survival [58]. In addition to the neurotrophin family, the neuropoietic cytokines, such as ciliary neurotrophic factor (CNTF) and leukemia inhibitory factor, are also considered members of neurotrophic factor family [32–34]. Recent findings have indicated that neurotrophic factors such as BDNF can serve as potential therapeutic candidates in neurodevelopmental disorders such as Rett syndrome and CDD [30, 31, 59, 60]. In this study, we examined the effects of a CNTF-derived peptidergic compound, P021, on neuronal maturation and survival in a human cellular model and in a mouse model of CDD. While treatment with P021 restored survival, proliferation, and maturation of a human cellular model of CDKL5 deficiency [20], it had very few effects on behavioral performance or neuroanatomical defects in *Cdkl5* KO mice that were treated either starting from a young age or in adulthood.

### Multiple deficits in a human neuronal model of CDKL5 deficiency are reverted by treatment with P021

To facilitate the identification of new relevant disease modifying molecules, we have recently generated an in vitro platform of a human cellular model of CDKL5 deficiency that reproduces defects that have already been reported in vivo in the mouse model of the disease, such as reduced neuronal survival, proliferation and differentiation, as well as alterations in the GSK3β signaling pathway [20]. Using this in vitro model, we found that treatment with P021 restored all these cellular defects that are due to the lack of CDKL5 expression. Since one mechanism behind the beneficial effects of CNTF on brain function is its ability to downregulate GSK3β activity [61], the effects of P021 on the neuronal model of CDKL5 deficiency may be ascribed, at least partially, to its inhibitory effect on GSK3β. This is in agreement with previous evidence that indicates that pharmacological inhibition of GSK3β activity improves, or even restores, neuronal survival and maturation in in vitro models of CDD [25, 56]. In parental SH-SY5Y cells, treatment with P021 had no effect on neuronal proliferation and survival, and a very scarce effect on neuronal maturation. This is in accordance with the failure of P021 to inhibit GSK3β activity in parental cells. The promising effect of P021 in vitro prompted us to investigate the effect of this compound on brain development in vivo.

The best recovery of defective phenotypes in SH-*CDKL5*-KO cells was obtained with the P021 dose of 10 µM, while lower concentrations (1 µM) were not effective in recovering the CDKL5-null neuroblastoma phenotype. The P021 dose used in this study was at least tenfold higher than that used by Kazim and colleagues on primary neuronal cultures to induce BDNF expression [52]. Although we do not currently have a justification for why SH-*CDKL5*-KO cells are less responsive to P021 than primary neurons, we might hypothesize that neuroblastoma cells have lower CNTF receptor expression levels, and consequently a reduced responsiveness to the CNTF signaling. However, we cannot exclude the possibility that the lack of CDKL5, by modifying/reducing the activation of intracellular CNTF signaling, causes a reduced responsiveness of SH-*CDKL5*-KO cells to P021, which could somehow justify the lack of the in vivo effect of P021 treatment at the tested doses.

### Treatment with P021 restored only some of behavioral defects in *Cdkl5* KO mice

*Cdkl5* KO mice exhibit multiple abnormal behaviors related to CDKL5 deficiency, including autistic-like behaviors, and defects in motor coordination and memory performance. We found that only some of the several CDD-related behavioral deficits are restored or improved in *Cdkl5* KO mice after chronic treatment (two and a half months) with P021, starting from the third postnatal week (P21). Specifically, we found that social and locomotor, as well as learning and memory deficits are not improved. Only a trend toward an improved anxiety-related behavior, assessed using the open field test, was found in P021-treated *Cdkl5* KO mice. Differently, the cataleptic behavior, a state characterized by a loss of control in the motor system which produces the appearance of “frozen” or motionless postures, was restored by P021 treatment. Considering that dopaminergic mechanisms underlie catalepsy and anxiety [62], and that CNTF has trophic effects for the dopaminergic neurons of the substantia nigra [63], we can speculate that P021, by affecting dopaminergic signaling, may selectively improve these defective behaviors. It should be noted that SH-SY5Y cells in both undifferentiated and differentiated states express a number of dopaminergic neuronal markers, such as dopamine transporter (DAT), as well as dopamine receptor subtypes (D2R and D3R) [64]. Their dopaminergic features might strengthen the hypothesis of a P021 trophic effect on CDKL5-null dopaminergic neurons, and justify the therapeutic efficacy of P021.

### Treatment with P021 did not restore neuroanatomical defects in *Cdkl5* KO mice

Unexpectedly, differently from what was observed in vitro, treatment with P021 was not able to promote proliferation (neurogenesis), neuronal survival (survival of hippocampal newborn granule and mature pyramidal cells), or neuronal maturation (spine maturation of hippocampal pyramidal neurons) in *Cdkl5* KO mice. This incongruity may be due to the fact that the in vitro model represents a simplified system that reveals molecular alterations that may be compensated in a complex and intricate in vivo brain network. Moreover, in contrast with the observation that neuroinflammation detected by microgliosis was ameliorated by P021 treatment [52, 65], treatment with P021 did not recover microglia activation in the Cdkl5-null brain. Since we did not find any effect of P021 treatment in *Cdkl5* KO mice, we hypothesized that the P021 dose used in the oral treatment protocol was not sufficient to restore the Cdkl5-related neuroanatomical defects, thus failing to lead to a normalization of behavioral phenotypes. Accordingly, we adopted an experimental protocol with a higher concentration of P021 (750 nmol/mouse), injected intraperitoneally (i.p.) every day for 30 days. Intraperitoneal administration was chosen because pharmacokinetic studies in rodents indicate that i.p. administration of small molecule pharmacological agents results in higher bioavailability, and in faster and more complete absorption compared to oral routes [66].

Even at this higher dose, P021 was not effective in improving neuroanatomical defects in *Cdkl5* KO mice, suggesting that in vivo activation of CNTF-dependent signaling is not sufficient to reverse defects that are due to the absence of Cdkl5. Initiation of the P021 treatment prenatally or for a more prolonged period than ~ 70 days could be required to achieve a significant increase in BDNF levels and rescue of autistic and cognitive behaviors.

## Conclusions

Although the exact mechanism of action of P021 associated with neuroprotection and neuroplastic changes is unknown, the in vivo properties of P021 have been attributed to the increase in BDNF expression [44], leading to activation of the PI3K-Akt signaling pathway that induces an inhibition of GSK3β activity via phosphorylation at Ser9 by Akt. Our finding that P021 treatment does not induce an increase in BDNF expression, while justifying the absence of treatment efficacy in the murine model of CDD, might suggest the presence of alterations in the mechanisms of CTNF-dependent response in the Cdkl5-null brain. However, previous findings showed that in the hippocampus of wild-type mice, P021 does not cause an increase in BDNF levels, and consequently does not affect behavior, in contrast with findings observed in a mouse model of Down syndrome [42]. The finding that treatment with P021 shows no advantages in normal animals is in line with the present evidence in *Cdkl5* KO mice, and suggests that P021 treatment may help brain development/function under some abnormal brain conditions such as Alzheimer’s disease (AD) [52] and Down syndrome (DS) [42], but not in the case of CDKL5 deficiency. However, we cannot exclude the possibility that gender or background specific differences could impact the results obtained in the male model of CDD, the *Cdkl5* -/Y mouse, compared to AD and DS mouse models [42, 52]. Indeed, while genetic background is similar among CDD, AD, and DS mice, all derived from crossbreeding with the C57BL/6 mouse strain (i.e., C57BL/6 J for *Cdkl5* KO mice; C57BL/6JEiJxC3Sn.BLiAFi for Ts65Dn mice, and C57BL/6 × 129/Sv for 3xTg-AD mice), the effects of P021 treatment in the mouse model of Alzheimer’s disease were evaluated exclusively in female mice, while a mixed female and male population of Ts65Dn mice were used as a model of Down syndrome. Since there are considerable differences between mice and humans, particularly regarding genetics, a more thorough understanding of the molecular mechanisms underlying P021 efficacy in human CDD cells *versus* mouse models will provide deeper insight into relevant disease mechanisms and into the actual therapeutic potential of P021 in CDD.

## Supplementary Information

Additional file 1: Supplementary figures. The Additional file 1 contains the following supplementary figures: Figure S1. Effect of P021 treatment on total GSK3β levels in SH- CDKL5 -KO cells; Figure S2. Effect of chronic oral P021 treatment on locomotor activity of Cdkl5 KO mice; Figure S3. Effect of chronic oral P021 treatment on BDNF levels and TrkB-PI3K-Akt-GSK3β signaling in Cdkl5 KO mice.

## Data Availability

Data sets generated during the current study are available from the authors upon reasonable request.

## Abbreviations

CDKL5: Cyclin-dependent kinase-like 5
CDD: CDKL5 deficiency disorder
CNTF: Ciliary neurotrophic factor
CNS: Central nervous system
AD: Alzheimer’s disease
DS: Down syndrome
AIF-1: Anti-allograft inflammatory factor 1
DCX: Doublecortin
DG: Dentate gyrus
GL: Granular layer
