# Supplementary material for: Effects of a ciliary neurotrophic factor (CNTF) small-molecule peptide mimetic in an in vitro and in vivo model of CDKL5 deficiency disorder
Source: J Neurodev Disord. 2024 Nov 26;16:65. doi: 10.1186/s11689-024-09583-4 (PMC11590213; doi:10.1186/s11689-024-09583-4)
Supplement: Supplementary file 1 — Additional file 1: Supplementary figures. The Additional file 1 contains the following supplementary figures: Figure S1. Effect of P021 treatment on total GSK3β levels in SH- CDKL5 -KO cells; Figure S2. Effect of chronic oral P021 treatment on locomotor activity of Cdkl5 KO mice; Figure S3. Effect of chronic oral P021 treatment on BDNF levels and TrkB-PI3K-Akt-GSK3β signaling in Cdkl5 KO mice. [file 11689_2024_9583_MOESM1_ESM.pdf]

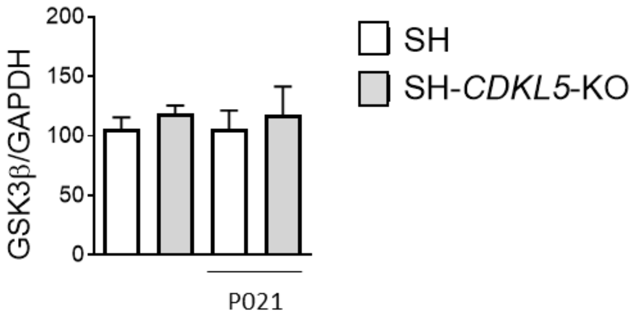

Figure S1. Effect of P021 treatment on total GSK3β levels in SH-*CDKL5*-KO cells.

Western blot analysis of total GSK3β levels in protein extracts from vehicle-treated (SH *n* = 5; SH-*CDKL5*-KO *n* = 5) and P021-treated SH-SY5Y (SH *n* = 3) and SH-*CDKL5*-KO (*n* = 4) cells. The histogram shows GSK3β protein levels normalized to GAPDH levels. Data are expressed as a percentage of vehicle-treated SH-SY5Y cells. Values are represented as means ± SEM. Fisher's LSD test after two-way ANOVA.

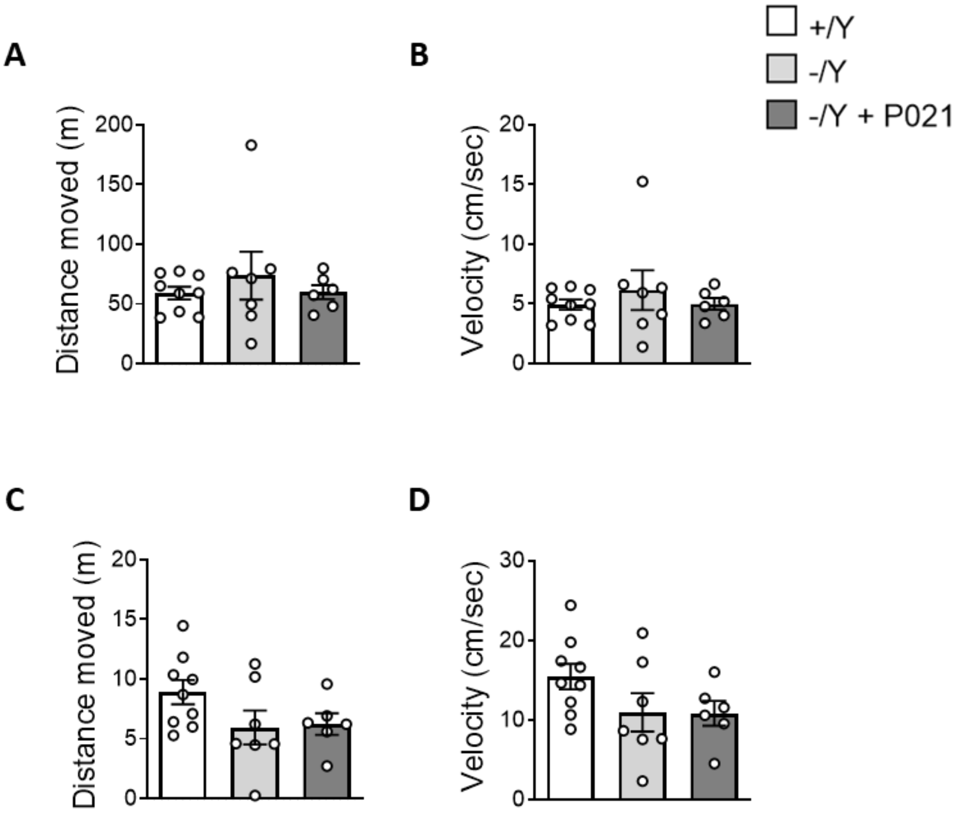

Figure S2. Effect of chronic oral P021 treatment on locomotor activity of *Cdkl5* KO mice.

A, B: Locomotor activity measured as total distance traveled (**A**) and mean velocity (**B**) during a 20-min open field test in orally vehicle-treated (+/Y *n* = 9, −/Y *n* = 7) and P021-treated (−/Y + P021 *n* = 6) *Cdkl5* male mice. **C, D:** Swimming locomotor activity in the Morris water maze, measured as total distance traveled (**C**) and mean velocity (**D**), during a 5-day learning period in vehicle-treated *Cdkl*5 −/Y and +/Y mice and in P021-treated −/Y *Cdkl5* mice as in A. Values are represented as means ± SEM. Fisher's LSD test after one-way ANOVA.

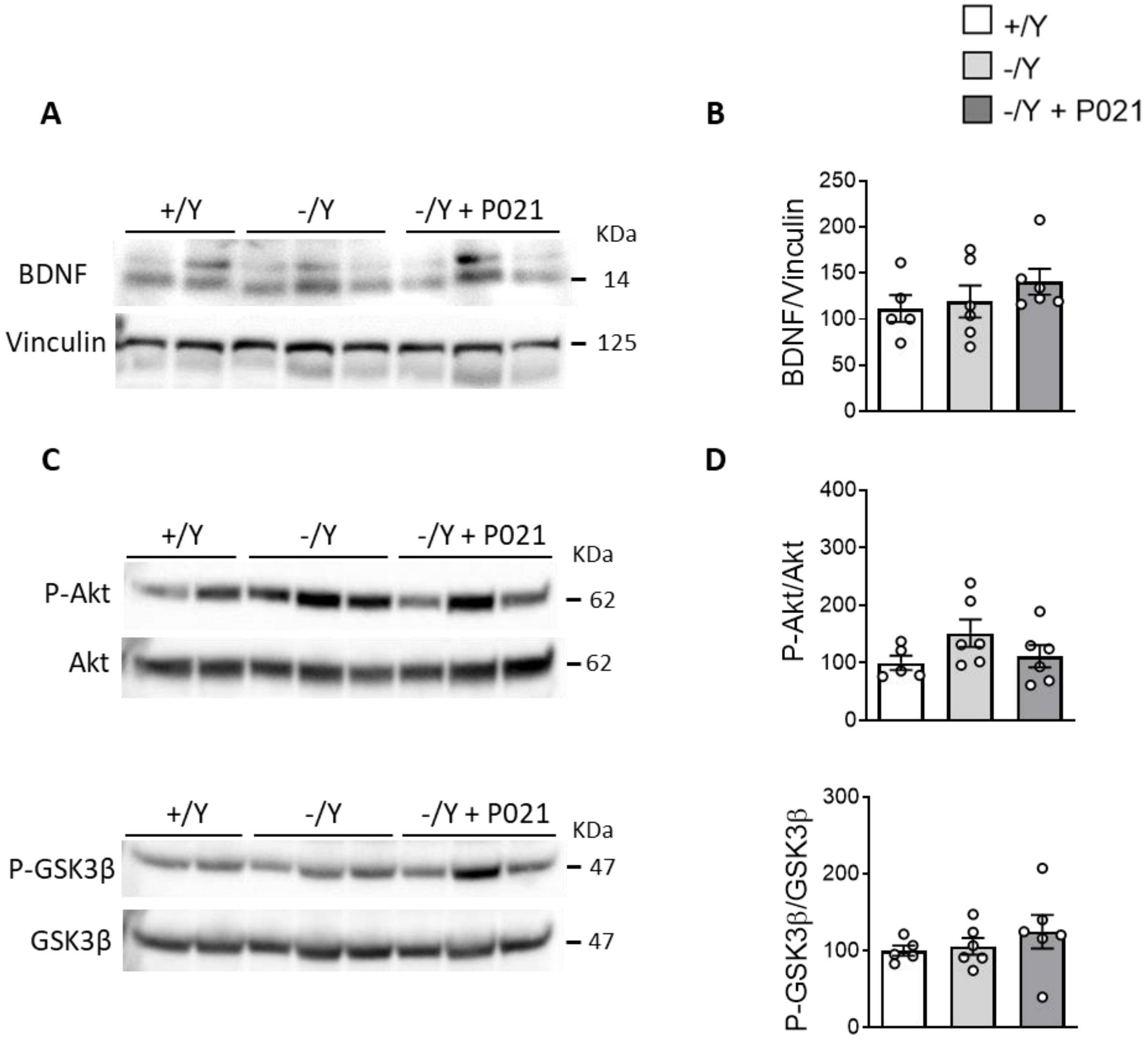

Figure S3. Effect of chronic oral P021 treatment on BDNF levels and TrkB-PI3K-Akt-GSK3β signaling in *Cdkl5* KO mice.

A,B: Western blot analysis of BDNF levels in hippocampal homogenates from vehicle-treated (+/Y $n = 5$, −/Y $n = 6$) and P021-treated (−/Y + P021 $n = 6$) *Cdkl5* male mice. The histogram in B shows mature BDNF protein levels normalized to Vinculin protein levels. Examples of immunoblots from multiple biological replicates of each experimental condition in A. **C,D:** Western blot analysis of P-Akt and P-GSK3β levels in hippocampal homogenates of mice as in B. Histograms in D show: P-Akt (ser473) protein levels normalized to total Akt levels (upper histogram), P-GSK3β (ser9) protein levels normalized to total GSK3β levels (lower histogram). Examples of immunoblots from biological replicates of each experimental condition in C. Data are expressed as a percentage of vehicle-treated wild-type (+/Y) mice. Values are represented as means ± SEM. Fisher's LSD test after one-way ANOVA.
